# Paxlovid accelerates cartilage degeneration and senescence through activating endoplasmic reticulum stress and interfering redox homeostasis

**DOI:** 10.1186/s12967-022-03770-4

**Published:** 2022-11-26

**Authors:** Keyu Kong, Yongyun Chang, Hua Qiao, Chen Zhao, Xuzhuo Chen, Kewei Rong, Pu Zhang, Minghao Jin, Jingwei Zhang, Huiwu Li, Zanjing Zhai

**Affiliations:** 1 Shanghai Key Laboratory of Orthopaedic Implants, Department of Orthopaedic Surgery, Shanghai Ninth People’s Hospital, Shanghai Jiaotong University School of Medicine, 639 Zhizaoju Road, Shanghai, 200011 People’s Republic of China; 2 Shanghai Key Laboratory of Stomatology, Department of Oral Surgery, College of Stomatology, Ninth People’s Hospital, Shanghai Research Institute of Stomatology, National Clinical Research Center of Stomatology, Shanghai Jiao Tong University School of Medicine, Shanghai, China

**Keywords:** Paxlovid, COVID-19, Osteoarthritis, Chondrocytes degeneration, Senescence

## Abstract

**Background:**

The COVID-19 pandemic has become a huge threat to human health, infecting millions of people worldwide and causing enormous economic losses. Many novel small molecule drugs have been developed to treat patients with COVID-19, including Paxlovid, which block the synthesis of virus-related proteins and replication of viral RNA, respectively. Despite satisfactory clinical trial results, attention is now being paid to the long-term side effects of these antiviral drugs on the musculoskeletal system. To date, no study has reported the possible side effects, such as osteoarthritis, of Paxlovid. This study explored the effects of antiviral drug, Paxlovid, on chondrocyte proliferation and differentiation.

**Methods:**

In this study, both in vitro and in vivo studies were performed to determine the effect of Paxlovid on chondrocyte degeneration and senescence. Furthermore, we explored the possible mechanism behind Paxlovid-induced acceleration of cartilage degeneration using transcriptome sequencing and related inhibitors were adopted to verify the downstream pathways behind such phenomenon.

**Results:**

Paxlovid significantly inhibited chondrocyte extracellular matrix protein secretion. Additionally, Paxlovid significantly induced endoplasmic reticulum stress, oxidative stress, and downstream ferroptosis, thus accelerating the senescence and degeneration of chondrocytes. In vivo experiments showed that intraperitoneal injection of Paxlovid for 1 week exacerbated cartilage abrasion and accelerated the development of osteoarthritis in a mouse model.

**Conclusions:**

Paxlovid accelerated cartilage degeneration and osteoarthritis development, potentially by inducing endoplasmic reticulum stress and oxidative stress. Long-term follow-up is needed with special attention to the occurrence and development of osteoarthritis in patients treated with Paxlovid.

**Supplementary Information:**

The online version contains supplementary material available at 10.1186/s12967-022-03770-4.

## Background

The number of SARS-CoV-2 infections worldwide continues to rise rapidly, as the recently emerged Omicron variant shows enhanced transmissibility compared to that of the previous variants [1–4]. Although a number of vaccines have been widely administered in many countries [5], some patients still develop severe symptoms leading to increase in hospitalizations and number of deaths. Traditional antiviral drugs, such as hydroxychloroquine [6] and lopinavir/ritonavir [7], have shown unsatisfactory anti-SARS-CoV-2 effects in clinical trials. However, in addition to symptomatic treatments, such as hemodynamic support and ventilation support, a number of novel SARS-CoV-2 antiviral drugs, including remdesivir [8], molnupiravir [9], and Pfizer's oral antiviral drug Paxlovid [9], have recently received Emergency Use Authorization from the FDA. Relative risk of hospitalization or death was reduced by 30% in a group treated with molnupiravir, according to the results of a Phase III clinical trial [10]. In addition, interim data from Pfizer's Phase II clinical trial showed that, among participants treated within 3 days of the onset of COVID-19 symptoms, the Paxlovid group had an 89% reduction in the risk of COVID-19-related hospitalization or death from any cause compared to that of the placebo group [11, 12].

Remdesivir and molnupiravir are nucleotide analogues, which integrate into nascent viral RNA strands, resulting in premature termination of RNA replication [9, 13]. Paxlovid consists of two components, with PF-07321332 (nirmatrelvir) being the main active component that can inhibit the activity of precursors of SARS-CoV-2 main protease, thus inhibiting the synthesis of virus-related proteins and viral replication. Nirmatrelvir is primarily metabolized by the CYP3A4 enzyme while ritonavir is an inhibitor of both HIV-1 protease and CYP3A4, thus ensuring the maintenance of sufficient blood concentrations of nirmatrelvir [9]. Recommended dosage of Paxlovid by FDA was 300 mg nirmatrelvir and 100 mg ritonavir twice a day for five consecutive days [14]. Paxlovid, a popular antiviral drug used to treat SARS-CoV-2, has been reported to cause adverse events, such as taste disorders, diarrhea, hypertension, and myalgia, in clinical trials [14]. Given the inhibition of CYP3A4 by ritonavir, drug interactions may be another source of serious adverse reactions [15, 16].

Recently, increasing attention has been paid to the effects of SARS-CoV-2 infection on bone metabolism [17, 18]. However, to date, no study has reported whether the use of these novel antivirals will initiate or accelerate the progression of degenerative diseases of the musculoskeletal system. Osteoarthritis, one of the most common degenerative joint disease, is the leading cause of disability worldwide [19]. It is characterized by the degeneration of articular cartilage, osteophyte formation, and osteosclerosis of subchondral bone and synovitis [20, 21]. Chondrocytes are responsible for secreting extracellular matrix, which comprises the articular surface. Aberrant mechanical conditions, inflammation in the microenvironment, and senescence are the main causes of chondrocyte degeneration and extracellular matrix degradation [22, 23]. In recent years, the role of endoplasmic reticulum stress (ER stress) has emerged in the pathogenesis of osteoarthritis [24]. When exposed to conditions such as hypoxia or nutrient deprivation, unfolded proteins will accumulate in the ER and the unfolded protein response (UPR) is activated to restore ER homeostasis. However, continuous and uncontrollable UPR also initiates cell death signaling and abolishes tissue homeostasis locally [25].

Ferroptosis is a form of cell death characterized by iron-dependent lipid peroxidation. A balance of labile iron ion concentrations, reactive oxygen species (ROS), and antioxidant enzymes regulates the production and elimination of peroxidized phospholipids [26, 27]. Oxidative stress and subsequent ferroptosis have been shown to be significantly involved in the development of osteoarthritis, and many therapies targeting ferroptosis and oxidative stress have achieved satisfactory outcomes in patients with osteoarthritis [28, 29]. Nuclear factor erythroid 2-related factor 2 (Nrf2) is the key regulator for the maintenance of redox homeostasis. Recent studies have revealed its connection with glucose and glutamine metabolism. Nrf2 activation redirects glucose and glutamine into anabolic pathways, which contributes to tumor proliferation and resistance to chemotherapies [30, 31]. In addition, redox signaling coordinates with ER stress to determine the cell fate since increased ROS levels can aggravate ER stress. Activating transcription factor 4 (Atf4), a downstream transcription factor of ER stress, can further strengthen the transcription activity of Nrf2 and increase the expression of antioxidant enzymes [32]. ER stress is composed of three independent signaling pathways: Ire1α-Xbp1s, Perk-eIF2α-Atf4, and Atf6 pathways.

At present, whether the interplay of redox signaling and ER stress participates in the effects of Paxlovid on chondrogenic differentiation and progression of osteoarthritis remains unknown. Therefore, this study aimed to investigate the effects of Paxlovid, current FDA approved anti-SARS-CoV-2 drug, on chondrogenic differentiation in vitro and osteoarthritis progression in vivo. The findings are expected to attract increased attention to the occurrence of osteoarthritis in patients with COVID-19 treated with Paxlovid.

## Methods

### Animals and surgical procedures

C57BL/6 J mice were purchased from the animal center of Shanghai Ninth People’s Hospital, and animal experimental plans were approved by the ethics committee of the hospital (SH9H-2022-A95-1). Twenty-four 12-week-old male mice were divided randomly into four treatment groups: Sham, Paxlovid, DMM (destabilization of the medial meniscus), and DMM + Paxlovid. For mice receiving the DMM surgery, we anesthetized them with isoflurane. Following exposure of the right knee joint, we bluntly dissected the fat pad and transected the medial meniscotibial ligament to enable destabilization of the medial meniscus. 150 mg/kg of nirmatrelvir and 50 mg/kg of ritonavir dissolved in 10% dimethyl sulfoxide (DMSO) and 90% corn oil were injected intraperitoneally every day in the third week after DMM surgery in Paxlovid and DMM + Paxlovid treated groups. The Sham and DMM groups were intraperitoneally administered the same volume of phosphate buffered saline (PBS). Mice were sacrificed four weeks after surgery, and both knee joints were collected and fixed in 75% ethanol after being treated for 48 h in 4% paraformaldehyde (PFA) for future micro-computed tomography (micro-CT) and histologic analysis.

### Micro-CT scanning

After fixation, right knee joints of the mice were scanned using a micro-CT scanner (Skyscan 1072; Skyscan, Belgium) at a resolution of 10.5 μm with 55 k Vp source and 145 μ Amp current. Imaging profiles were analyzed and remodeled in CT-An and CT-Vox software (Bruker, Germany) to analyze the subchondral bones and osteophytes.

### Histology and immunofluorescence staining

For histologic and immunofluorescence analyses, the prefixed knee joints were decalcified in 10% ethylenediaminetetraacetic acid for 2 weeks, then embedded into paraffin. Serial tissue sectioning of 5 μm thickness in a sagittal plane was performed and stained withand safranine O/fast green to evaluate cartilage degeneration and clefts. OARSI score was evaluated according to the standards of Sophocleous [33].

For immunofluorescence staining, the knee joint sections were de-paraffinized in xylene and standard alcohol gradients and then washed with PBS three times. Then, the sections were incubated with an antigen retrieval buffer for 15 min and washed with PBS three times. Then, the sections were incubated with an anti-fluorescence quencher for 5 min and blocked with Ultra V block for 15 min at room temperature to block nonspecific antibody binding sites. Anti-Col2a1 (ab34712, 1:200) and anti-p21 (ab188224, 1:1000) primary antibodies were purchased from Abcam, United Kingdom, while anti-Ddit3 (15,204-1-AP, 1:200) and anti-Sod1 (10,269-1-AP, 1:200) primary antibodies were purchased from Proteintech, United States. The sections were incubated with the corresponding antibodies at 4℃ overnight and incubated with corresponding secondary antibodies the next day. The sections were then washed with PBS and the nucleus was stained with DAPI. Images were obtained randomly using a Zeiss DM4000B microscope, and the ImageJ software was used to carry out quantitative analysis by counting the positive cells in each visual field.

### Cell culture and reagents

We isolated murine chondrocytes from 3-week-old C57BL/6 J mice. After dissecting the femoral head of the mouse, we carefully isolated a layer of cartilage from the femoral head and spliced it into small pieces. After digestion in 0.25% trypsin for 30 min, these cartilage pieces were digested in 0.2% collagenase type 2 for 4 h in a cell incubator. The next day, the cells were centrifuged, and chondrocytes were cultured in Dulbecco’s modified eagle medium (DMEM) with 4.5 g glucose/L, 10% fetal bovine serum (FBS), and 1% penicillin–streptomycin (Gibco, Thermo Fisher Scientific, Waltham, MA, United States).

We purchased the immortalized mouse chondrocyte cell line ATDC5 from the Cell Bank of the Chinese Academy of Sciences (Shanghai, China). The ATDC5 cells were cultured in DMEM with 4.5 g glucose/L, 5% FBS, and 1% penicillin–streptomycin. Cells were incubated in a humid environment at 37℃ with 5% CO_2_. Paxlovid, GSK2606414, and N-acetylcysteine (NAC) were purchased from MCE (New Jersey, United States) and dissolved in DMSO. We ensured that the final DMSO concentration in the medium used for cell culture was less than 0.1%.

### Micromass culture

Micromass culture was used to evaluate the collagen secretion ability of chondrocytes. After digestion, centrifugation, and suspension, a 1.5 × 10^7^ per mL ATDC5 cell suspension was prepared, and 10 μL of the suspension was added to the center of each well in a 24-well culture plate. After 2 h in the cell incubator, 1 mL of DMEM with 5% FBS and insulin-transferrin-selenium (ITS) (Gibco, Thermo Fisher Scientific, Waltham, MA, United States) was added to each well. Medium was added gently to avoid deformation of the micromass. Medium was replaced every other day for 7 days. After fixation in 4% PFA for 10 min, alcian blue and toluidine blue dyes were added to stain the extracellular matrix in the micromass.

### Cell proliferation and cytotoxicity

The effect of Paxlovid on the proliferation of chondrocytes was determined using the Cell Counting Kit 8 (CCK8). Chondrocytes were seeded into 96-well culture plate at a density of 6,000 ATDC5 cells per well and cultured with 100 μL DMEM supplemented with 5% FBS and 1% penicillin–streptomycin. Medium was replaced on the second day, and different concentrations of Paxlovid were added at 24, 48, and 72 h. At each timepoint, 100 μL of the medium containing 10% CCK8 buffer (Dojindo, Kumamoto, Japan) was added to the wells and incubated in a cell incubator for 2 h to avoid light exposure. Absorbance was read at the 450 nm wavelength using an Infinite M200 pro multimode microplate reader (Tecan Life Sciences, Switzerland).

### Cell cycle analysis

The cell cycle stage of the ATDC5 cells was analyzed using flow cytometry. After being cultured with different concentrations of Paxlovid for 24 h, the ATDC5 cells were digested, centrifuged, and suspended with 1 mL PBS three times. Pre-cooled 75% ethanol was used to fix the cells at 4 ℃ overnight. After fixation, the cells were centrifuged and suspended with 1 mL PBS three times the next day. Then, 5 μg/mL DAPI (Beyotime, Nanjing, China) was used to stain the cells placed on ice and protected from light exposure for 15 min. The cell cycle phase profile was collected on a BD Fortessa system (BD Bioscience, USA) and analyzed using FlowJo 10 (BD Bioscience, USA) to calculate the proportion of cells in different phases.

### Senescence-associated-β-galactosidase (SA-β-gal) staining

Cellular senescence was detected using SA-β-gal staining. Murine chondrocytes were cultured with different concentrations of Paxlovid for 7 days in a 24-well-plate. After fixation with 4% PFA for 10 min, a SA-β-gal staining kit (Beyotime, Nanjing, China) was used to stain the senescent cells at 37 ℃ overnight. Images were taken randomly with a Zeiss microscope, and the ImageJ software was used for quantitative analysis.

### ROS detection

Intracellular ROS was detected using a DCFH-DA probe (1:1000; S0033S; Beyotime Biotechnology, China). Briefly, the ATDC5 cells were plated in confocal dishes (Cellvis, CA, USA), then cultured with different concentrations of Paxlovid for 24 h. Then, the cells were incubated in serum-free culture medium containing 10 μM DCFH-DA for 20 min at 37 ℃. After that, the cells were stained with DAPI for nuclear fluorescence, then washed with PBS three times. Fluorescent images were captured via confocal microscopy (Zeiss DM4000B, Leica). The ImageJ software was used for semi-quantitative analysis of ROS-positive cells.

### FerroOrange staining

A FerroOrange kit (F374, Dojindo, Shanghai, China) was used to detect intracellular Fe^2+^. After the Paxlovid treatment, the ATDC5 cells were washed with PBS and stained with DAPI for nuclear fluorescence, then washed with PBS three times. Then, the cells were incubated with FerroOrange working solution (1 μmol/L) for 30 min. Fluorescent images were captured via confocal microscopy (Zeiss DM4000B, Leica).

### Protein extraction and Western Blot analysis

After incubation with different concentrations of Paxlovid for 24 h in a six-well plate, 200 μL of the RIPA lysis buffer (Beyotime, Nanjing, China) with a 1% cocktail of protease and phosphatase inhibitors (Thermo Fisher Scientific, Waltham, MA, United States) was added to each well containing the treated cells. The whole lysis process, performed on ice, was completed in 30 min. After, the cells were centrifuged at 12,000 × *g* for 15 min, the bicinchoninic acid assay was used to detect the protein concentration of the supernatant. 5X SDS-sample loading buffer was then added to the supernatant, and the protein was boiled at 99℃ for 10 min.

For western blot analysis, 20 μg protein per well was loaded onto SDS-PAGE gels and then transferred to a polyvinylidene fluoride membrane (Millipore, Merck, Germany). The membrane was blocked using 5% non-fat milk for 1 h at room temperature to block nonspecific antigen sites. After, the membrane was rinsed with tris-buffered saline and Tween 20 three times, it was incubated with the corresponding primary antibody overnight at 4 ℃. The membrane was incubated with secondary fluorescence antibodies for 2 h at room temperature, and the membrane was visualized using the Odyssey V3.0 image scanner (Li-COR. Inc., Lincoln, NE, USA). Quantitative measurements were made by measuring the gray value of each strip. Anti-Sox9 (ab185966, 1:1000), anti-CollagenII (ab34712, 1:1000), anti-p16 (ab211541, 1:2000), and anti-p21 (ab188224, 1:1000) primary antibodies were purchased from Abcam. Anti-β-actin (4970, 1:2000), anti-Xbp1s (40,435, 1:1000), anti-Atf4 (11,815, 1:1000), anti-Bip (3177, 1:1000), anti-Elf2α (9722, 1:1000), anti-phospho-Elf2α (3398, 1:1000), anti-Ddit3 (2895, 1:1000), and anti-Ire1α (3294, 1:1000) primary antibodies were purchased from CST, Boston, United States. Anti-Ho1 (10,701-1-AP, 1:1000) and anti-Sod1 (10,269-1-AP, 1:1000) primary antibodies were purchased from Proteintech. The secondary antibody was anti-rabbit IgG (H + L; DyLight™ 800 4 × PEG conjugate; Abcam).

### Quantitative real-time PCR (qRT-PCR)

For qRT-PCR, total RNA was extracted from the treated ATDC5 cells using a total RNA kit (R6812-01HP, Omega Bio-tek Ink., Norcross, GA, United State). 1000 ng of cDNA was prepared according to the protocols provided by the cDNA synthesis kit (Takara, Shiga, Japan) and diluted to 200 μL. A total (10 μL) of 3.2 μL ddH_2_O, 1 μL cDNA, 0.4 μL upstream primer, 0.4 μL downstream primer, and 5 μL SYBR premix were mixed and added to each well of a 384-well plate. qRT-PCR was performed using an ABI 7500 Sequencing Detection System (Applied Biosystems, Foster City, CA, USA). All primer sequences for the target genes are listed in Table 1. Gene expression levels were calculated using the 2^−△△CT^ method, and data were presented as fold increase compared to controls.

**Table 1:** Primers used in the qRT-PCR assay

Gene	Organisms	Forward (5′-3′)	Reverse (5′-3′)
Gapdh	Mus musculus	GGCAAGTTCAACGGCACAG	CGCCAGTAGACTCCACGACAT
Col2a1	Mus musculus	GCTACACTCAAGTCACTGAACAACCA	TCAATCCAGTAGTCTCCGCTCTTCC
Sox9	Mus musculus	CGTGGACATCGGTGAACTGAG	GGTGCTGCTGATGCCGTAAC
P16	Mus musculus	GGTCACACGACTGGGCGATT	GCACCGTAGTTGAGCAGAAGAG
P21	Mus musculus	GCCTGGTTCCTTGCCACTTCTT	ATTACGGTTGAGTCCTAACTGCCATC
P53	Mus musculus	CTCCAGCTACCTGAAGACCAAGAAG	GCAGAGACCTGACAACTATCAACCTAT
Atf3	Mus musculus	GGTCGCACTGACTTCTGAGG	CTCTGGCCGTTCTCTGGA
Atf4	Mus musculus	TGGCGAGTGTAAGGAGCTAGAAA	TCTTCCCCCTTGCCTTACG
Atf6	Mus musculus	AGAGGCAGCACACGCATTCC	TGATGGTCAGCAGGAGCAGAGA
Chop	Mus musculus	CATACACCACCACACCTGAAAG	CCGTTTCCTAGTTCTTCCTTGC
Ire1α	Mus musculus	CTGGCGAGAAGCAGCAGACTT	CCACCACAGGAGAGGCATAGTT
Bip	Mus musculus	CCTCATCGGACGCACTTGGAAT	GCTTGTCGCTGGGCATCATTG
Xbp1s	Mus musculus	GAGTCCGCAGCAGGTG	GTGTCAGAGTCCATGGGA
Fgf21	Mus musculus	CAGGGGTCATTCAAATCCTG	AGGAATCCTGCTTGGTCTTG

### RNA sequencing

The ATDC5 cells (3 × 10^5^) were seeded in a six-well-plate and stimulated with Paxlovid (none in control group and 60 μM in Paxlovid group) for 24 h. Total RNA was extracted from the cells using a total RNA kit (R6812-01HP, Omega Bio-tek Ink., Norcross, GA, United State), according to the manufacturer’s protocol and then analyzed by RNA sequencing performed by Wuhan Huada Gene Technology Co., Ltd. (China). Kyoto Encyclopedia of Genes and Genomes (KEGG) pathway enrichment analysis, Gene ontology (GO) enrichment analysis, gene set enrichment analysis (GSEA), and heat maps using mRNA relative expression as transcripts per kilobase million were performed using the Mybgi platform (Wuhan Huada Gene Technology, https://mybgi.bgi.com/tech/ login).

### Statistical analysis

Statistical analyses were conducted using SPSS version 19.0 for Windows (SPSS Inc., Chicago, IL.). All data are representative of more than three independent experiments unless otherwise indicated. For data with a normal distribution, comparison between two groups was analyzed using unpaired Student’s t tests. Comparisons between three or more groups was analyzed using one-way ANOVAs and Student-Newman-Keuls post hoc tests. For ranked data, Mann–Whitney and Kruskal–Wallis tests were used to compare between two groups or multiple groups. All statistical charts were designed using GraphPad Prism 8 (GraphPad Software Inc, San Diego, CA, USA). A P value < 0.05 was considered significant.

## Results

### Effects of Paxlovid on cell viability of ATDC5 cells

**Fig. 1:**
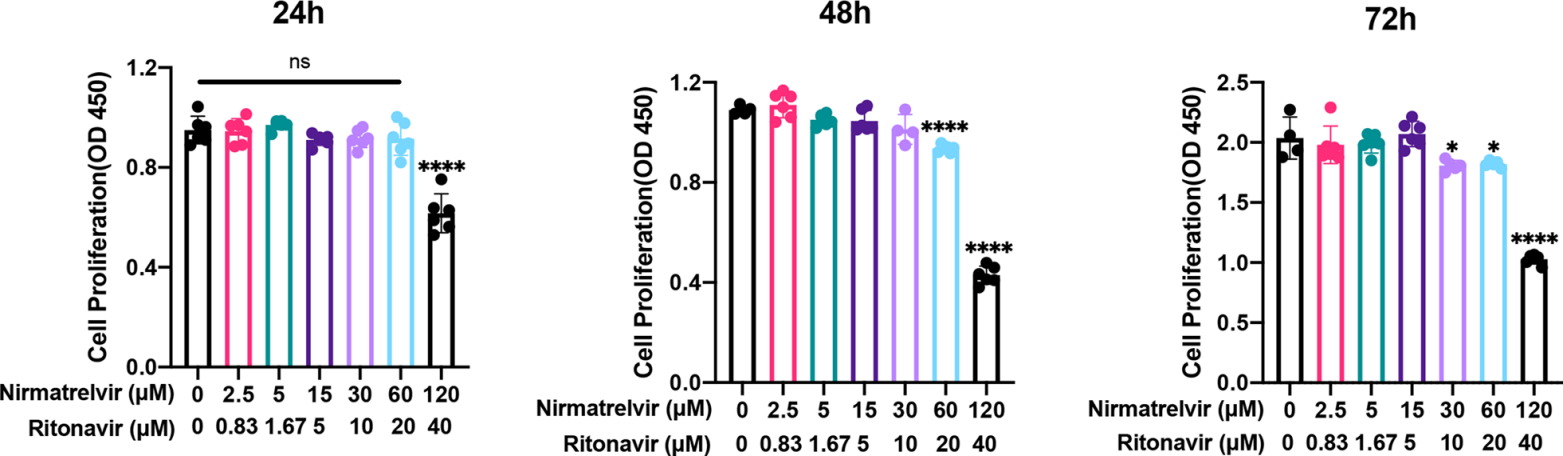
Cell viability of the ATDC5 cells treated with different concentrations of Paxlovid at different timepoints *P < 0.05, **P < 0.01, ***P < 0.001, and ****P < 0.0001, compared to the control group

The SARS-CoV-2 EC_50_ (effective concentration to inhibit 50% cell growth) for Paxlovid was 77.9 [9] nM. To test its cytotoxicity on ATDC5 cells, we performed the CCK8 test 24, 48, and 72 h after incubating the cells with different concentrations of Paxlovid. We observed that 120/40 μM Paxlovid exerted evident cytotoxicity and hampered cell viability significantly (Fig. 1). Lower concentrations of Paxlovid inhibited cell proliferation in a concentration-dependent manner, which indicated an effect on cell senescence. Thus, 60 / 20 μM Paxlovid were used as maximum concentrations in further chondrogenic differentiation experiments.

### Paxlovid hinders chondrogenic differentiation, extracellular matrix secretion, and cell senescence

**Fig. 2:**
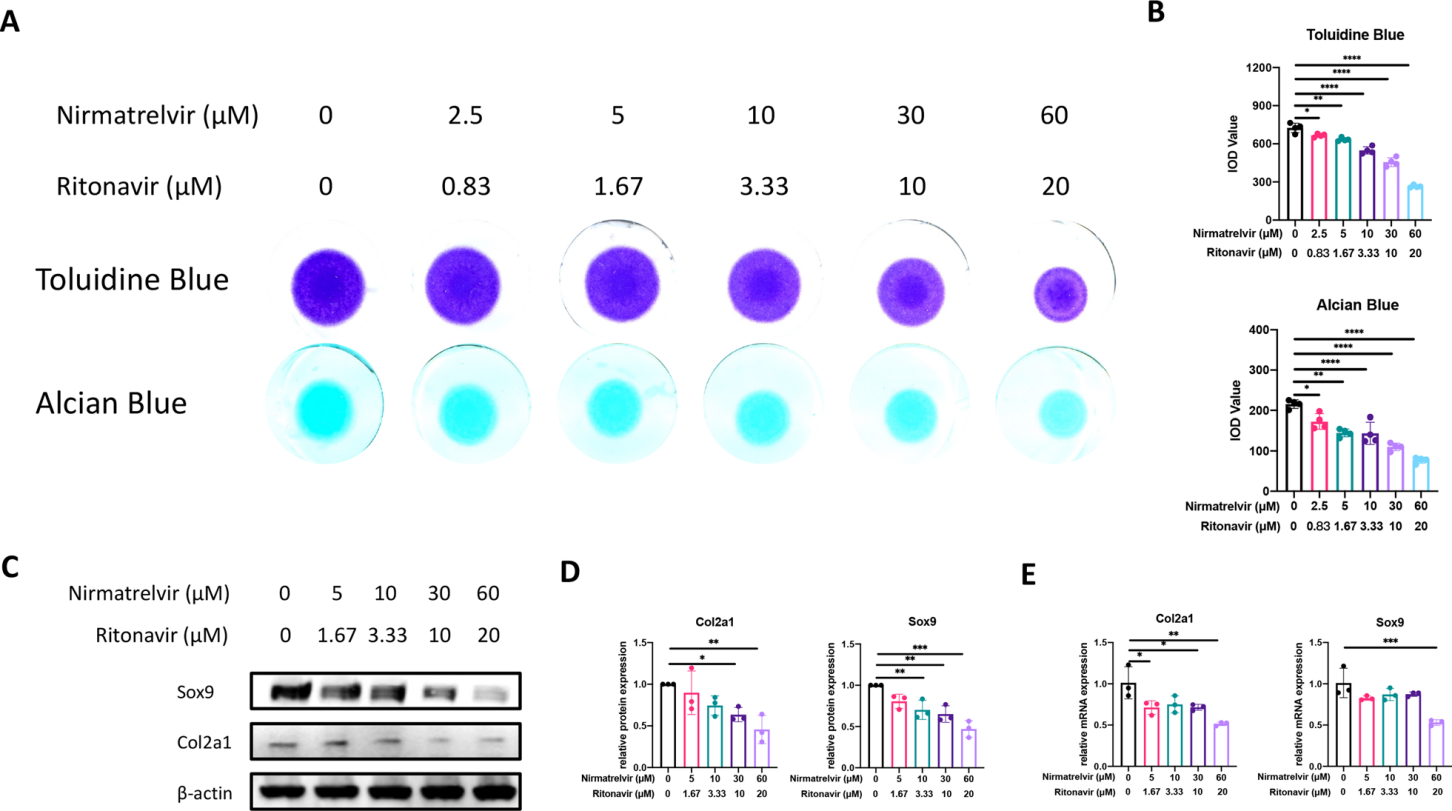
Effect of Paxlovid on the ATDC5 chondrogenic differentiation ability. **A**, **B** Alcian blue and toluidine blue staining of micromass cultures of the ATDC5 cells and quantitative analysis, which reflects the effect of Paxlovid on extracellular matrix protein secretion. **C**, **D** Expression of chondrogenic-related genes when cells are treated with a concentration gradient of Paxlovid, with corresponding quantitative analysis. **E** mRNA expression of *Sox9* and *Col2a1* with and without Paxlovid treatment. *P < 0.05, **P < 0.01, ***P < 0.001, and ****P < 0.0001, compared to the control group

To explore the effects of Paxlovid on the accumulation of extracellular matrix proteins, such as collagen and proteoglycan, we cultured ATDC5 cells in a micromass manner under the stimulation of Paxlovid for 7 consecutive days to simulate COVID-19 treatment. Alcian blue staining results revealed that a concentration gradient of Paxlovid from 2.5/0.83 μM to 60/20 μM showed an evident inhibitory influence on matrix protein secretion in a concentration-dependent manner, as observed for both alcian blue and toluidine blue staining (Fig. 2A, B). To further verify its effect on chondrogenic differentiation, we performed qRT-PCR and western blot analyses to examine mRNA expression and protein expression, respectively, of chondrogenic genes, such as Sox9 and Col2a1 in ATDC5 cells. Results were consistent with the alcian blue and toluidine blue staining. 10 μM nirmatrelvir and 3.33 μM ritonavir triggered significant changes in gene expression after 24 h of treatment (Fig. 2C–E).

**Fig. 3:**
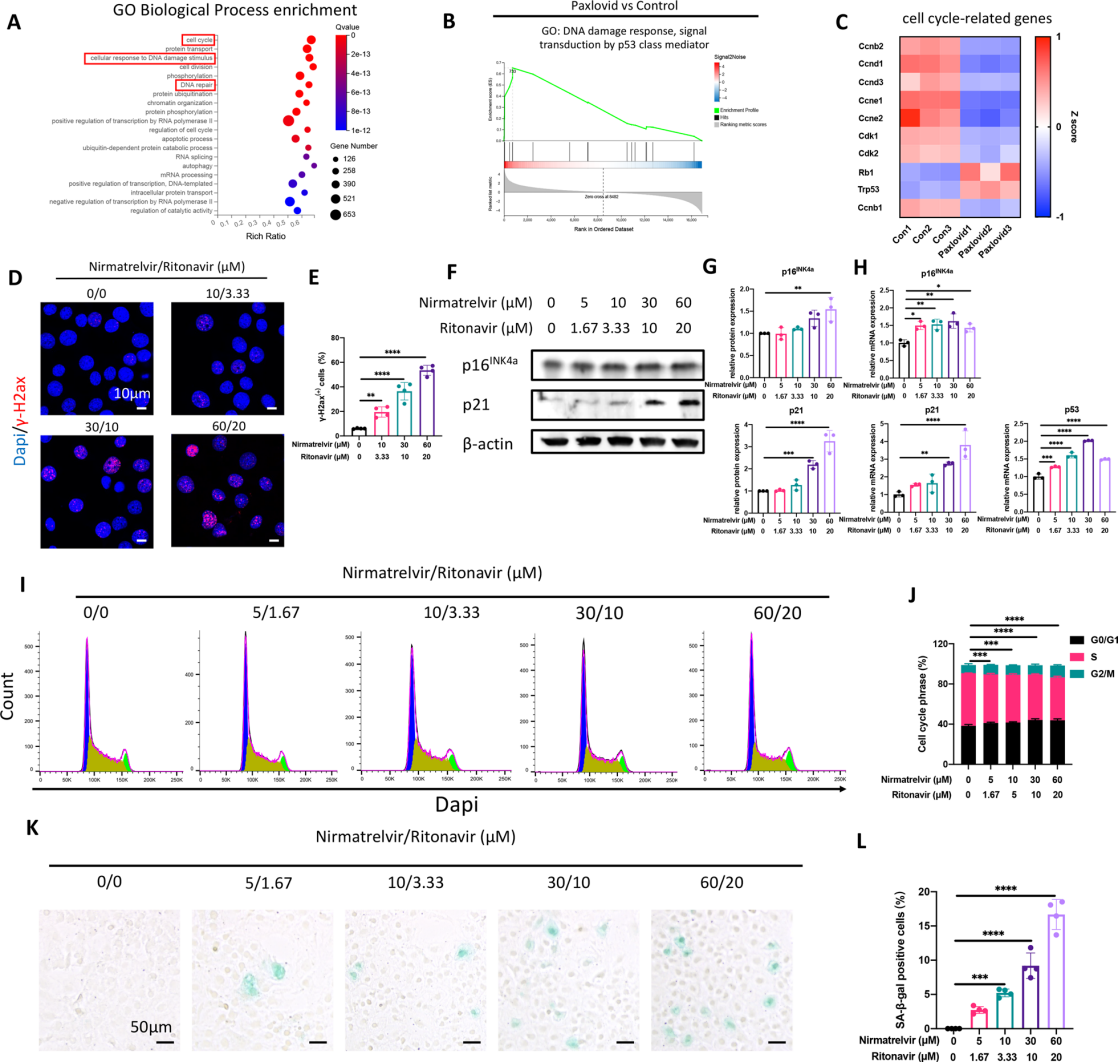
Paxlovid accelerates chondrocyte senescence through the DNA damage response. **A** Gene ontology (GO) biological process enrichment analysis of differentially expressed genes presented as a bubble chart. **B** Gene set enrichment analysis of genes involved in GO biological processes: DNA damage response and signal transduction by p53 class mediator. **C** Heatmap of mRNA expression of genes involved in the cell cycle. **D**, **E** Immunofluorescence of γ-H2AX and positive cell proportions when treated with different concentrations of Paxlovid. Scale bar: 10 μm. **F** mRNA expression of cell cycle-related genes p16^INK4a^, p21, and p53 when treated with different concentrations of Paxlovid. **G**, **H** Western blot with quantitative analysis and protein expression of p16^INK4a^, p21, and p53 after Paxlovid treatment. **I** Cell cycle analysis of the ATDC5 cells treated with Paxlovid. Blue, yellow and green parts represent G0/G1, S, and G2/M phases, respectively. (J) Quantitative analysis of the proportion of cells in different cell phases. **K**, **L** Senescence-associated-β-galactosidase staining of mouse chondrocytes treated with Paxlovid and corresponding quantitative analysis. Scale bar: 50 μm. *P < 0.05, **P < 0.01, ***P < 0.001, and ****P < 0.0001, compared to control group

After confirming the inhibitory effects of Paxlovid on chondrogenic differentiation, we further explored the mechanisms behind such phenomena. RNA sequencing was performed, and subsequent GO biological process enrichment analysis and GSEA both indicated a significant role of cell senescence and DNA damage response (Fig. 3A–C). Immunofluorescence of γ-H2AX, which is a biomarker of DNA damage and repair, showed that the DNA damage response and repair were activated under stimulation of different concentrations of Paxlovid (Fig. 3D, E). Cell senescence is characterized by cell cycle arrest, which is when cells initiate their DNA repair program after sensing DNA damage at certain checkpoints. When treated with Paxlovid, the expression of p16^INK4a^, p21, and p53 mRNA and proteins was elevated (Fig. 3F–H), which indicated that the cell cycle was delayed under the influence of Paxlovid treatment in ATDC5 cells. Cell cycle analysis was also performed using flow cytometry, and the FlowJo software was used to calculate the proportion of cells in different phases of cell division (Fig. 3I, J). Results showed that the proportion of cells in synthesis (S) phase decreased when treated with Paxlovid, while the proportion of cells in GO/G1 and G2/M phases increased with the increase in Paxlovid concentrations. SA-β-gal staining results also indicated that a higher number of murine chondrocytes exhibited higher β-galactosidase activity, under a pH of 6.0 (Fig. 3J, K).

### Paxlovid triggers ER stress and disrupts redox homeostasis

**Fig. 4:**
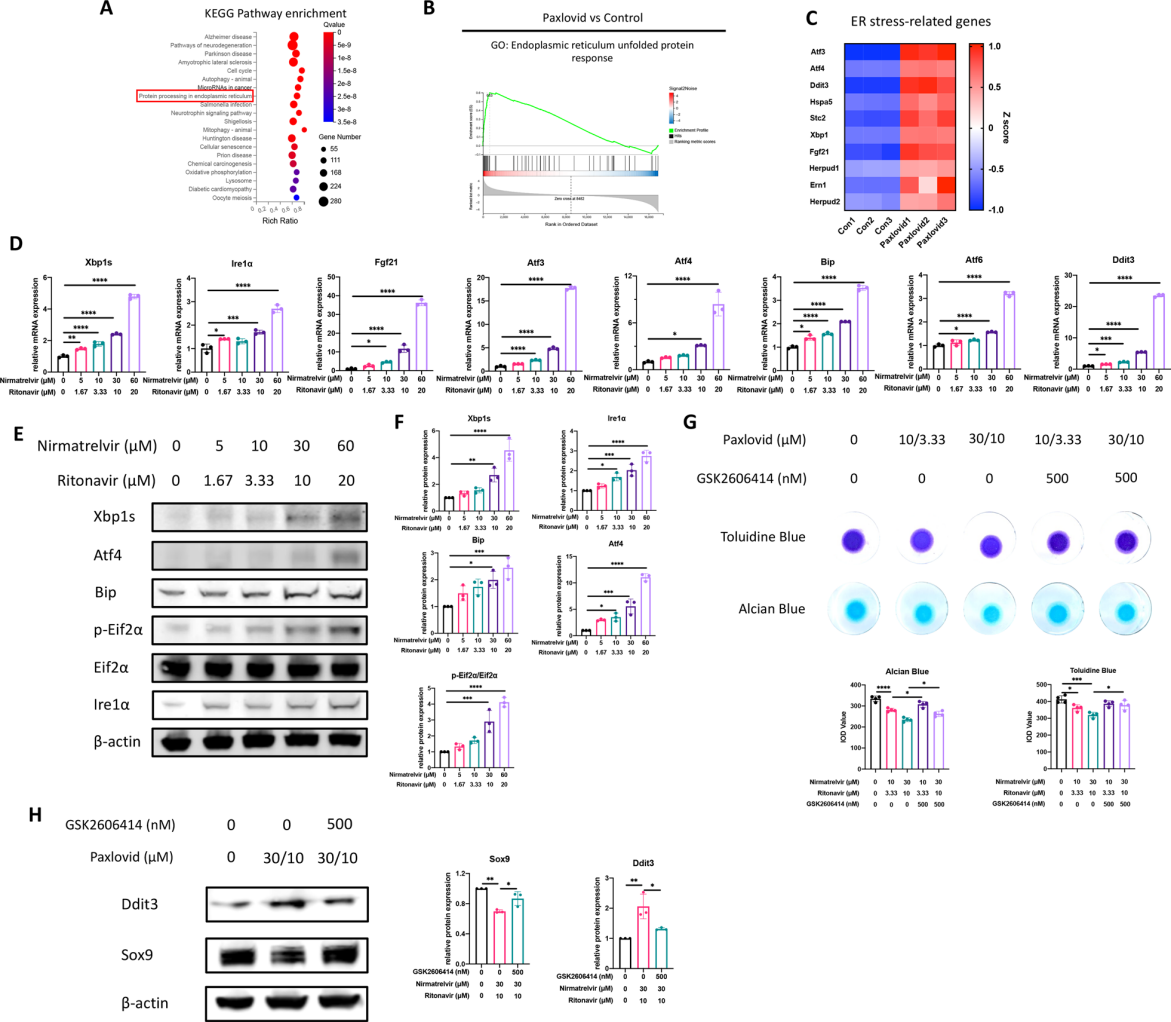
Paxlovid triggers evident ER stress in the ATDC5 cells. **A** Kyoto Encyclopedia of Genes and Genomes (KEGG) pathway enrichment analysis of differentially expressed genes presented as a bubble chart. **B** Gene set enrichment analysis of genes involved in the KEGG biological process: Endoplasmic reticulum unfolded protein response. **C** Heatmap of mRNA expression of ER stress-related genes. **D** mRNA expression of ER stress-related genes when treated with different concentrations of Paxlovid. **E**, **F** Western blot with quantitative analysis of ER stress-related genes after treatment with Paxlovid. **G** Alcian blue and toluidine blue staining show the rescue effect of GSK2606414 on extracellular matrix protein secretion. **H** Western blot results reveal the rescue effect of GSK2606414 on the protein expression of Ddit3 and Sox9. *P < 0.05, **P < 0.01, ***P < 0.001, and ****P < 0.0001

In addition to cell cycle arrest, ER stress and related pathways were also enriched in the Paxlovid treated cells according to KEGG pathway enrichment analysis and GSEA results (Fig. 4A–C). This prompted us to examine the level of ER stress in ATDC5 cells when treated with Paxlovid. mRNA expression analysis indicated that all three independent signaling pathways of ER stress were activated with Paxlovid treatment (Fig. 4D). Western blot and related quantitative analysis (Fig. 4E, F) confirmed this activation of ER stress. In addition, ER-stress inhibitor GSK2606414 rescued the downregulation of *Sox9* expression and extracellular matrix secretion as confirmed by western blot, alcian blue staining, and toluidine blue staining (Fig. 4G, H).

**Fig. 5:**
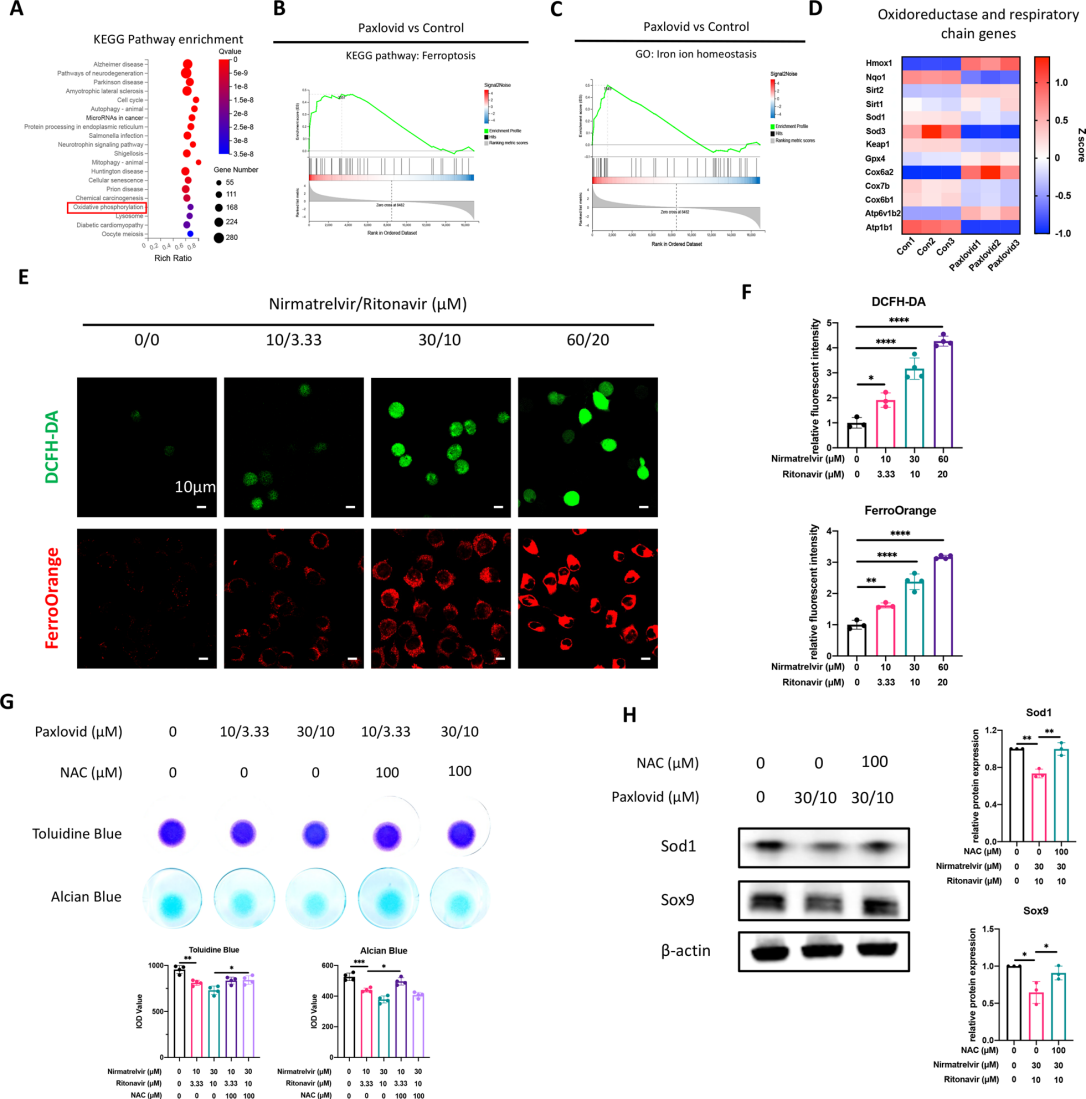
Paxlovid disrupts redox homeostasis and induces ferroptosis. **A** Kyoto Encyclopedia of Genes and Genomes (KEGG) pathway enrichment analysis of differentially expressed genes presented as a bubble chart. **B**, **C** Gene set enrichment analysis of genes involved in the KEGG pathways: ferroptosis and the gene ontology biological process: Iron ion homeostasis. **D** Heatmap of mRNA expression of genes involved in redox homeostasis. **E**, **F** Representative images of DCFH-DA-labelled reactive oxygen species and FerroOrange-labelled Fe.^2+^, with quantitative analyses. Scale bar: 10 μm. **G** Alcian blue and toluidine blue staining reveal the rescue effect of N-acetylcysteine (NAC) on extracellular matrix protein secretion. **H** Western blot analysis of Sod1 and Sox9 demonstrating NAC’s rescue effect on gene expression. *P < 0.05, **P < 0.01, ***P < 0.001, and ****P < 0.0001

Considering the evident ER stress and responses to DNA damage, we speculated that disrupted redox homeostasis occurred when cells were treated with Paxlovid. RNAseq results revealed that the oxidative phosphorylation pathway was enriched when cells were treated with Paxlovid (Fig. 5A). Ferroptosis and iron ion homeostasis were also interrupted (Fig. 5B, C). A heat map of related gene expression indicated that the expression levels of antioxidant enzymes and components of the oxidative respiratory chain were accordingly elevated or reduced (Fig. 5D). To directly observe redox and iron ion homeostasis in the ATDC5 cells, DCFH-DA and a FerroOrange kit were used to detect intracellular ROS and Fe^2+^. Representative images and semi-quantitative statistics all showed increased oxidative stress levels and Fe^2+^ levels intracellularly (Fig. 5E, F). Furthermore, NAC and ferrostatin-1 (Fer-1), which are ROS and ferroptosis inhibitors, respectively, were used to rescue the cartilage degeneration caused by Paxlovid (Fig. 5G, H, Additional file 1: Fig. S1). The expression of essential genes in oxidative stress and ferroptosis, *Sod1* and *Ho1*, was rescued when ATDC5 cells were incubated with NAC and Fer-1. In addition, downregulation of *Sox9* expression was also rescued under these conditions, thus verifying the involvement of redox homeostasis and ferroptosis in Paxlovid-induced chondrocyte degeneration.

### *Short-term exposure of Paxlovid accelerates progression of osteoarthritis *in vivo

**Fig. 6:**
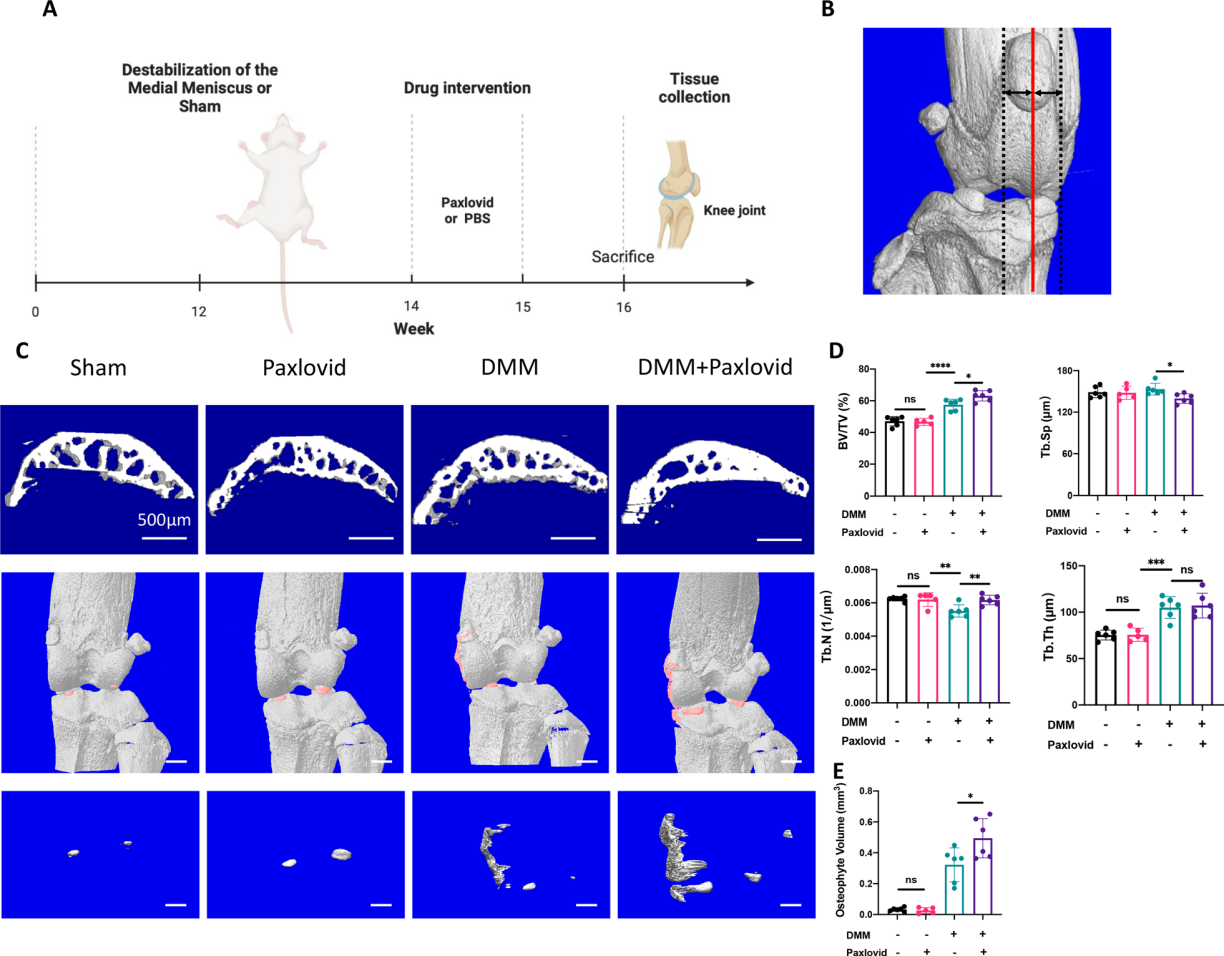
Paxlovid accelerates subchondral bone sclerosis and osteophytes formation in vivo. **A** A timeline of mouse experiments and their related interventions, created with BioRender.com **B** Strategy of subchondral bone reconstruction. The red line is perpendicular to the articular surface and passes through the midpoint of the medial tibial plateau. **C** Representative images of sagittal planes of subchondral bones and osteophytes in different treatment groups. Scale bar: 500 μm. **D** Quantitative analysis of micro-computed tomography scans: BV/TV, percent bone volume; Tb.N, trabecular number; Tb.Th, trabecular thickness; Tb.Sp, trabecular separation. N = 6, 6, 5, and 6 biologically independent joint samples of the Sham, Paxlovid, DMM, and DMM + Paxlovid groups, respectively. *P < 0.05, **P < 0.01, ***P < 0.001, and ****P < 0.0001

In order to explore the effects of short-term exposure to Paxlovid on articular cartilage in vivo, C57B6/J mice with either a sham operation or DMM were intraperitoneally administered with 150 mg nirmatrelvir and 50 mg ritonavir per kilogram every day for 1 week. An illustration and timeline of all relevant interventions used are depicted in Fig. 6A. After tissue collection, micro-CT scans were performed using all the knee joints, after which we performed reconstruction and quantitative analysis of the subchondral bones and osteophytes. We reconstructed the sagittal planes of subchondral bone in the middle level of the medial tibial plateau (Fig. 6B) to directly illustrate enhanced osteosclerosis in the DMM+Paxlovid treated group. Representative images and quantitative analysis of structural indices all showed that short exposure to Paxlovid accelerates the progression of osteoarthritis as more osteophytes were observed in Paxlovid+DMM group than those in the other groups (Fig. 6C, D).

**Fig. 7:**
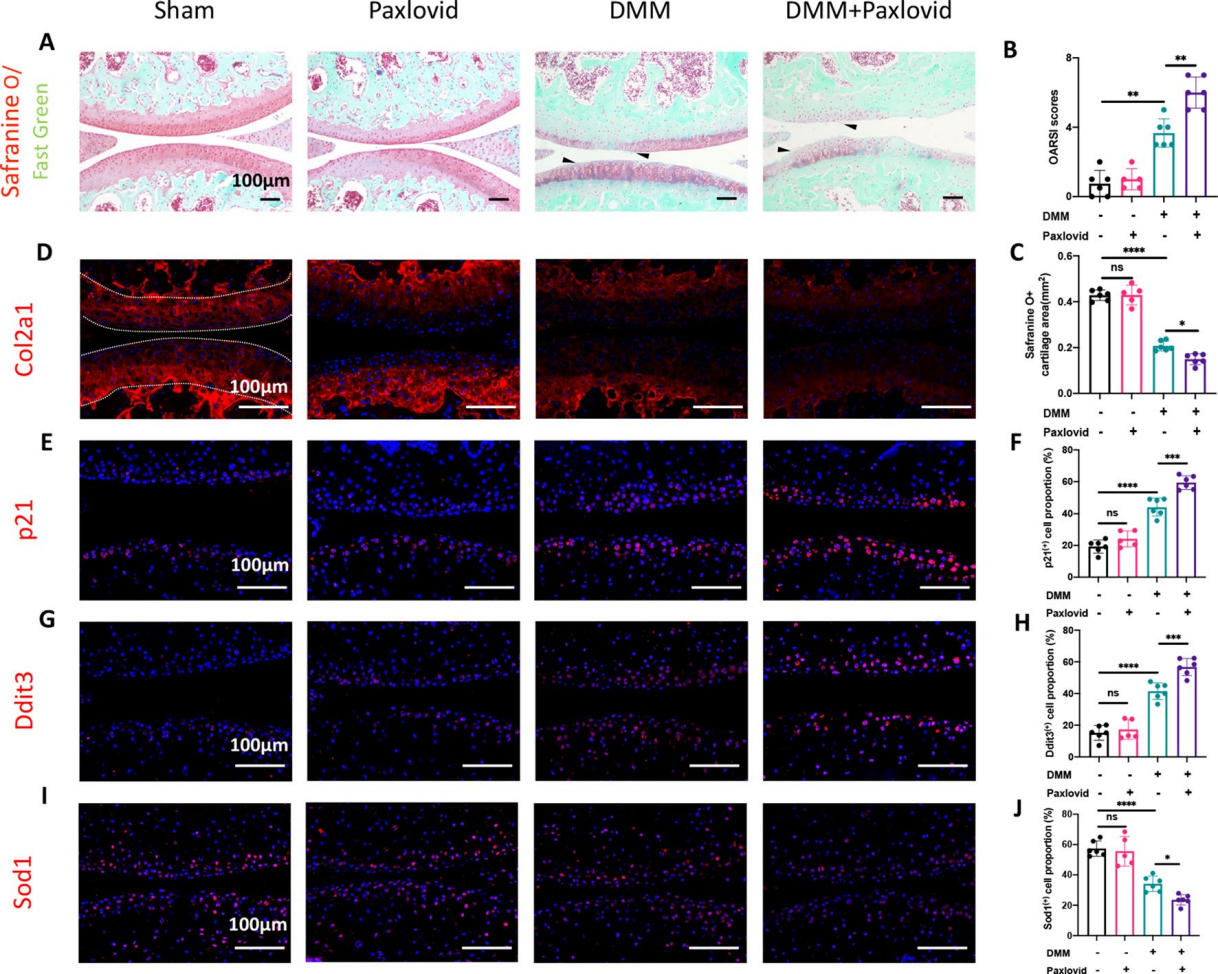
Paxlovid accelerates cartilage degeneration and senescence in vivo. **A**–**C** Fast green/ Safranine O staining of the knee joint, and quantitative analysis of the OARSI score and proportion of Safranine O positive cartilage area. The black triangle depicts superficial abrasion of cartilage. The OARSI score was calculated as the sum of separate OARSI scores of cartilages in the femoral and tibial parts. Scale bar: 100 μm. **D**–**J** Immunofluorescence of Col2a1, p21, Ddit3, and Sod1 in articular cartilage, and related quantitative analysis of the proportion of immunofluorescence-positive cells. Scale bar: 100 μm. *P < 0.05, **P < 0.01, ***P < 0.001, and ****P < 0.0001

**Fig. 8:**
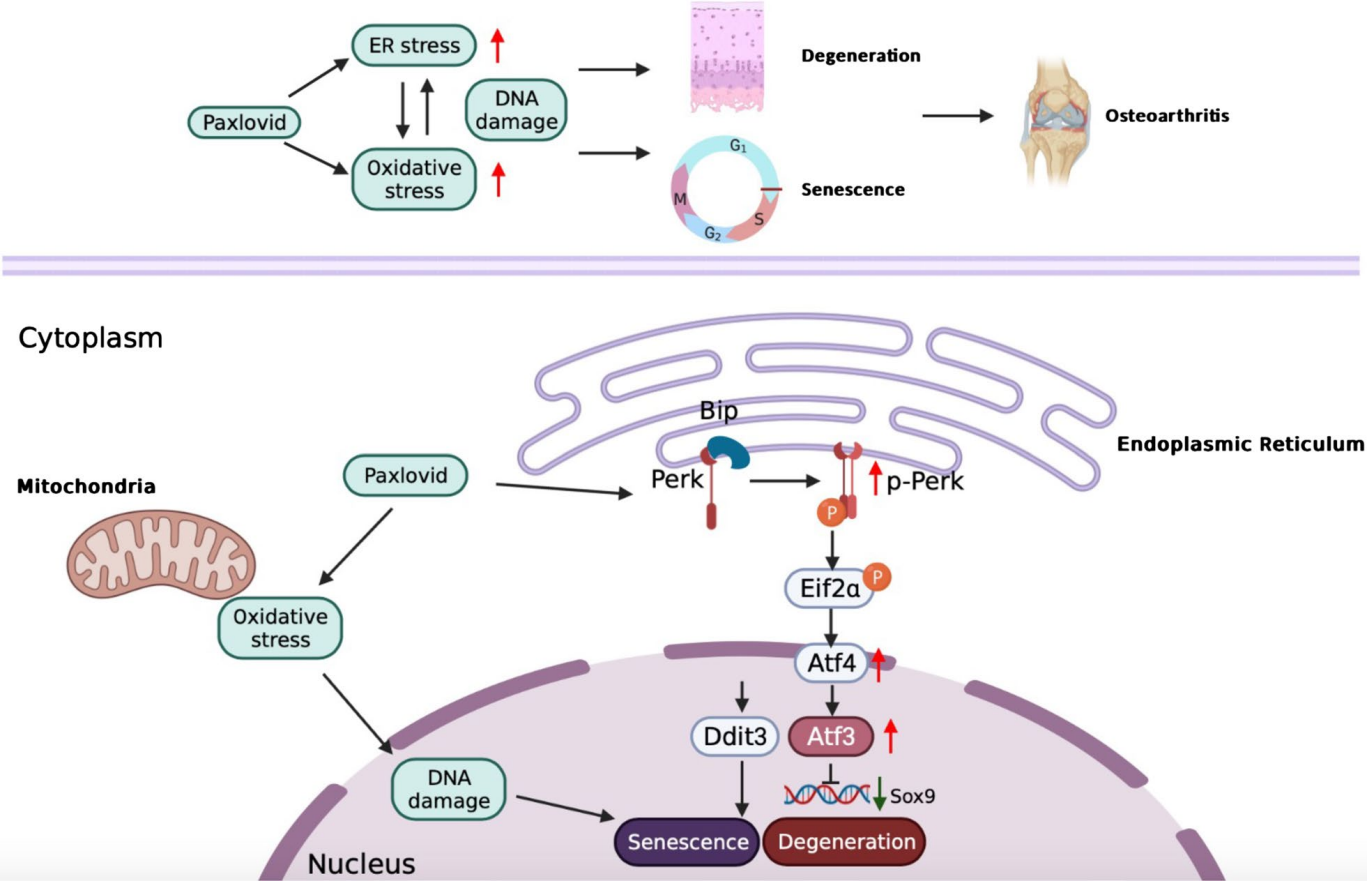
The role of Paxlovid in cartilage degeneration and its possible molecular mechanisms. A schematic chart created with BioRender.com

Histologically, fast green/ safranine O staining were used to evaluate the severity of cartilage abrasion in osteoarthritis. Cartilage abrasion was more serious and less Safranine O-positive cartilage was retained in the DMM + Paxlovid group than those in the other groups (Fig. 7A–C). Degeneration and senescence of chondrocytes in vivo were further verified by immunofluorescence analysis of Col2a1 and p21 (Fig. 7D–F). Immunofluorescence and semi-quantitative analysis of Ddit3 and Sod1 positive cells did show activation of ER stress and oxidative stress after Paxlovid treatment (Fig. 7G–J). A schematic chart was provided to illustrate the effect of Paxlovid on osteoarthritis and its mechanisms behind (Fig. 8).

## Discussion

At present, there are two main options under consideration for antiviral therapy for SARS-CoV-2 [13]. The first neutralizes the RBD area of the spike protein using monoclonal antibodies to inhibit it from binding to the ACE2 receptor [34], and the other involves small molecule drugs that interfere with virus replication. Recent research has found that more than 30 mutations exist in the spike protein of Omicron BA.1, which makes it capable of evading most anti-RBD neutralizing antibodies [35]. However, small molecule drugs, such as Paxlovid and molnupiravir, are still effective against Omicron BA.2. Kawaoka et al. [1] showed that nirmatrelvir and molnupiravir could inhibit the proliferation of Omicron BA.2 in the lower respiratory tract of hamsters. Considering the current prevalence of the Omicron mutant, humans are expected to coexist with Omicron and receive antiviral small molecule drugs for the foreseeable future. Therefore, it is of great significance to explore the long-term effects of SARS-CoV-2 infection and antiviral drugs on not only the respiratory system but also all the other systems.

Many studies have focused on the impact of Omicron infection on musculoskeletal diseases [17, 18]. Inflammation can influence bone remodeling, and researchers have reported that chronic obstructive pulmonary disease, asthma, and cystic fibrosis can disrupt bone metabolism and lead to pathological bone loss [36, 37]. In an established golden Syrian hamster model infected with SARS-CoV-2, significant multifocal loss of the bone trabeculae was found in the long bone and lumbar spine only 4 days after infection. This bone loss may be caused by the accumulation of circulating proinflammatory cytokines and activated osteoclast differentiation. Gao et al. [17] found that SARS-CoV-2 can also directly infect bone marrow macrophages through non-canonical receptor Nrp1 rather than through ACE2, which in turn inhibits their differentiation into osteoclasts. These studies suggest that SARS-CoV-2 has the potential to cause complications that so far have been neglected, suggesting that we will need to follow-up on patients with COVID-19 for a longer duration to determine the impact of the virus on target organs outside the respiratory system.

Till date, no literature has reported whether novel small molecule antiviral drugs pose similar side effects on the musculoskeletal system. To the best of our knowledge for the first time our study showed that Paxlovid can trigger ER stress and oxidative stress in chondrocytes, which gives rise to chondrocyte senescence and hampers chondrogenic differentiation and extracellular matrix secretion. Upon Paxlovid treatment, disrupted redox homeostasis and compensatory elevation of antioxidants coordinate with ER stress to accelerate the degeneration and senescence of chondrocytes. Our in vivo experiments also showed that in a mechanical instability model, the use of Paxlovid can aggravate the cleft of cartilage and calcified tissue generation. These results suggest that patients receiving Paxlovid treatment also need long-term follow-up to determine its impact on the development of osteoarthritis.

Drug safety currently focuses on the use of Paxlovid in special populations and on drug interactions, and previous studies [14] have shown that Paxlovid has no teratogenic effect on newborns. To the best of our knowledge for the first time our study investigated osteoarthritis as a side effect of anti-SARS-CoV-2 drugs. Our data revealed that 10 μM nirmatrelvir with 3.33 μM ritonavir was enough to evidently affect the gene expression associated with cartilage differentiation, senescence, and ER stress after a short 24 h exposure. After long-term exposure for 7 days, 2.5 μM nirmatrelvir with 0.67 μM ritonavir significantly reduced the level of collagen secreted by micromass cultured chondrocytes, which indicates that Paxlovid can interfere with chondrogenic differentiation in a concentration dependent manner. According to the present the pharmacokinetic data of Paxlovid, the maximal plasma concentration of nirmatrelvir is 2.21 μg/mL and area-under-the-plasma concentration versus time curve is 23.01 μg^**.**^hr/mL after a single dose of 300 mg nirmatrelvir and 100 mg ritonavir in healthy individuals [14, 38]. This is consistent with the effective concentration observed in our study. However, the knee cartilage is nourished by synovial fluid in the joint capsule and the local concentration of Paxlovid in synovial fluid is unknown. Therefore, further studies should explore the local drug concentration in patients. In addition, it is reasonable to speculate that Paxlovid also accelerates cell senescence and ER stress in other tissues considering its systemic circulation. A cohort of patients treated with Paxlovid ought to be established to monitor its long-term effects on the other systems, such as the cardiovascular system.

In vitro results showed that after a 7-day exposure, low concentration of Paxlovid was sufficient to reduce matrix protein secretion by chondrocytes. At present, Pfizer recommends Paxlovid twice a day for 5 consecutive days after infection [14]. Even if the symptoms are not significantly relieved, the medication duration is not recommended to be prolonged. To determine whether such medication frequency and duration have similar destructive effects on knee cartilage, the duration and frequency of Paxlovid administration recommended by Pfizer was used for mice following DMM surgery. Histological analysis supported that the administration of Paxlovid for 1 week accelerates the degeneration of cartilage. It is thus suggested that patients with COVID-19, especially those with knee osteoarthritis, may face a high risk of disease progression and further deterioration.

This study has some limitations. Firstly, there is no study reporting the Paxlovid concentration present locally in synovial fluid of the knee joint, which makes it difficult to determine whether the actual Paxlovid concentration would be sufficient to exert an effect on chondrocytes. Secondly, the ideal in vivo model would have both SARS-CoV-2 infection and osteoarthritis with Paxlovid treatment. In contrast, our animal model received Paxlovid treatment without SARS-CoV-2 infection. Animal models simulating the actual condition should be used to determine whether SARS-CoV-2 infection and Paxlovid treatment have a synergistic effect on the occurrence and development of arthritis. Finally, this study only addressed the phenomenon of Paxlovid triggering cartilage senescence and degeneration through ER stress, oxidative stress, and downstream ferroptosis, aggravating the development of arthritis, but we did not explore the specific molecular mechanism behind this aggravation. Further research is needed to elucidate the mechanistic relationships between Paxlovid, ER stress, and oxidative stress. Recently, several researches have demonstrated that Nrf2 also regulates glutamine metabolism reprograming, which contributes to cancer malignant phenotypes such as resistance to chemotherapy. Whether metabolism reprogramming also plays a role in Paxlovid-induced oxidative stress seems to be a promising theme to study [39, 40].

## Conclusions

Our in vitro and in vivo studies revealed that the novel anti-SARS-CoV-2 drug Paxlovid triggered ER stress, oxidative stress, and ferroptosis in chondrocytes, thus inducing DNA damage, hindering chondrogenic differentiation, and accelerating the progression of osteoarthritis. Therefore, we speculate that patients with COVID-19 treated with Paxlovid, especially those with preexisting osteoarthritis, may face an increase in risk of disease progression and further deterioration. Clinical studies are needed to further explore and support these results.

## Supplementary Information

**Additional file 1: Figure S1.** Ferroptosis inhibitor ferrostatin-1 rescues chondrocyte degeneration induced by Paxlovid. (A, B) Alcian blue and toluidine blue staining and corresponding quantitative analysis demonstrating the rescue effects of Fer-1 on extracellular matrix protein secretion. (C) Western blot analysis of Ho1 and Sox9 protein expression following treatment with Fer-1 demonstrating the rescue effect of Fer-1 on protein expression.

## Data Availability

The datasets used and/or analysed during the current study are available from the corresponding author on reasonable request.

## Abbreviations

ER stress: Endoplasmic reticulum stress
UPR: Unfolded protein response
ROS: Reactive oxygen species
Atf4: Activating transcription factor 4
Nrf2: Nuclear factor erythroid 2-related factor 2
NAC: N-acetylcysteine
PBS: Phosphate buffered saline
PFA: Paraformaldehyde
Micro-CT: Micro-computed tomography
DMEM: Dulbecco’s modified eagle
FBS: Fetal bovine serum
SA-β-gal: Senescence-associated-β-galactosidase
qRT-PCR: Quantitative real-time PCR
KEGG: Kyoto encyclopedia of genes and genomes
GO: Gene ontology
GSEA: Gene set enrichment analysis
