# Supplementary figures and images for: Paxlovid accelerates cartilage degeneration and senescence through activating endoplasmic reticulum stress and interfering redox homeostasis
Source: J Transl Med. 2022 Nov 26;20:549. doi: 10.1186/s12967-022-03770-4 (PMC9701426; doi:10.1186/s12967-022-03770-4)

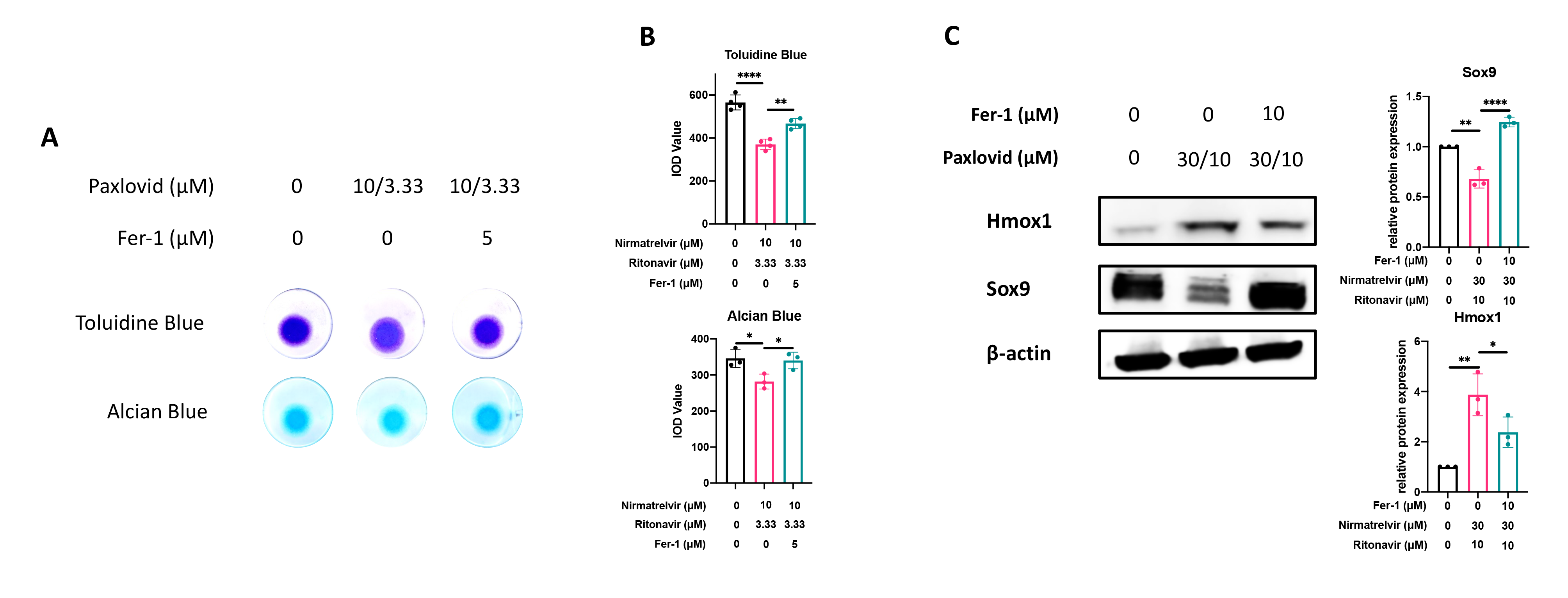

Supplement: Supplementary file 1 — Additional file 1: Figure S1. Ferroptosis inhibitor ferrostatin-1 rescues chondrocyte degeneration induced by Paxlovid. (A, B) Alcian blue and toluidine blue staining and corresponding quantitative analysis demonstrating the rescue effects of Fer-1 on extracellular matrix protein secretion. (C) Western blot analysis of Ho1 and Sox9 protein expression following treatment with Fer-1 demonstrating the rescue effect of Fer-1 on protein expression. [file 12967_2022_3770_MOESM1_ESM.tif]
